# Reduced Prefrontal Cortex Activation in Children with Attention-Deficit/Hyperactivity Disorder during Go/No-Go Task: A Functional Near-Infrared Spectroscopy Study

**DOI:** 10.3389/fnins.2017.00367

**Published:** 2017-06-28

**Authors:** Shuo Miao, Junxia Han, Yue Gu, Xin Wang, Wenhong Song, Dongqing Li, Zhao Liu, Jian Yang, Xiaoli Li

**Affiliations:** ^1^Department of Neurology, Children's Hospital Affiliated to Capital Institute of PediatricsBeijing, China; ^2^State Key Laboratory of Cognitive Neuroscience and Learning, Beijing Normal UniversityBeijing, China; ^3^IDG/McGovern Institute for Brain Research, Beijing Normal UniversityBeijing, China; ^4^Institute of Electrical Engineering, Yanshan UniversityQinhuangdao, China; ^5^Department of Health Care, Children's Hospital Affiliated to Capital Institute of PediatricsBeijing, China

**Keywords:** functional near-infrared spectroscopy (fNIRS), attention deficit hyperactivity disorder (ADHD), children, response inhibition, prefrontal cortex (PFC)

## Abstract

**Objective:** Attention-deficit/hyperactivity disorder (ADHD) is one of the most common neuropsychiatric disorders in children and affects 3 to 5% of school-aged children. This study is to demonstrate whether functional near-infrared spectroscopy (fNIRS) can detect the changes in the concentration of oxygenated hemoglobin (oxy-HB) in children with ADHD and typically developing children (TD children).

**Method:** In this study, 14 children with ADHD and 15 TD children were studied. Metabolic signals of functional blood oxygen were recorded by using fNIRS during go/no-go task. A statistic method is used to compare the fNIRS between the ADHD children and controls.

**Results:** A significant oxy-HB increase in the left frontopolar cortex (FPC) in control subjects but not in children with ADHD during inhibitory tasks. Moreover, ADHD children showed reduced activation in left FPC relative to TD children.

**Conclusion:** Functional brain imaging using fNIRS showed reduced activation in the left prefrontal cortex (PFC) of children with ADHD during the inhibition task. The fNIRS could be a promising tool for differentiating children with ADHD and TD children.

## Introduction

Attention-deficit/hyperactivity disorder (ADHD) is one of the most common neuropsychiatric disorders in children and affects 3 to 5%of school-aged children. The ADHD is mainly characterized by age-inappropriate symptoms of hyperactivity, inattentiveness, and impulsivity (American Psychiatric Association, 2013). These primary symptoms can be identified in children with ADHD during early elementary school years (Mucina, 2005). Furthermore, children with ADHD often develop comorbidities, including oppositional defiant disorder, antisocial behavior, substance abuse, and problems associated with conduct and learning later in life (Klassen et al., 2004; Wehmeier et al., 2010). Cognitive functioning is mildly impaired in this disorder (Sergeant et al., 2002). Particularly, the ADHD affects response inhibition, which is the ability to inhibit inappropriate thoughts and actions. Several studies found that inhibitory dysfunction is a key neurophysiological defect of ADHD (Durston et al., 2003; Smith et al., 2006; Bledsoe et al., 2010), and prefrontal cortex (PFC) is one of the most important region that highly influences response inhibition (Schmitz et al., 2006; Zang et al., 2006; Kana et al., 2007).

According to the cognitive model of Barkley, response inhibition involves three interrelated processes: (1) inhibition of an initial pre-potent response, (2) stopping of an ongoing response or delayed responding, and (3) limiting interference or distractibility during delay periods (Barkley, 1997). Go/no-go task is a classic neuropsychological tasks extensively used in clinical setting to assess response inhibition (Casey et al., 1997; Smith et al., 2006; Fang et al., 2010; Monden et al., 2012a). During this task, prepotent tendency is inhibited to execute a response. This inhibition may only occur at response-selection or execution stages (Rubia et al., 2001; Xiao et al., 2012). The overlap of stimulus or response leads to other forms of interference (Rubia et al., 2001; Wager et al., 2005).

Functional near-infrared spectroscopy (fNIRS) can measures changes in concentrations of oxygenated, deoxygenated, and total hemoglobin (oxy-HB, deoxy-HB, and total-HB) in brain hemodynamics by measuring the absorption of near-infrared light (usually in the range of 700–1,000 nm) projected through the scalp (Liao et al., 2013). fNIRS provides an indirect measure of neural activity based on changes in blood oxygenation due to metabolic processes within the cortex (Vanderwert and Nelson, 2014). Thus, we can assess the brain activation of ADHD children during neuropsychological tests using fNIRS. fNIRS has many advantages, such as noninvasiveness, non-radiative property, and insensitivity to motion artifacts; the fNIRS also provides data with high temporal resolution in comparison with fMRI (Quaresima et al., 2012).

Several researchers used fNIRS to investigate differences in PFC activation during response inhibition tasks (such as go/no-go test) between children with ADHD and matched typically developing children (TD children). Children with ADHD showed diminished PFC activation compared with TD children. However, the localization of inhibitory-associated activation within the frontal cortex is inconsistent among previous studies that employed fNIRS and go/no-go task. Monden (Monden et al., 2012a) used fNIRS to study children with ADHD executing response inhibition tasks; the results showed decreased level of activation of the right inferior frontal gyrus/middle frontal gyrus. In the study of Fangyue (Fang et al., 2010), children with ADHD were asked to perform inhibitory tasks; fNIRS results indicated that during the go/no-go task, children with ADHD showed weak activation in the left PFC. Conversely, in the study of Inoue (Inoue et al., 2012), children with ADHD showed significantly reduced activation in the bilateral frontal areas compared with TD children during no-go condition that requires inhibition.

In this study, we evaluated the activation of children with ADHD and TD children in the PFC during go/no-go task through fNIRS. We assume that brain activity will be altered in patients with ADHD in contrast to controls in PFC.

## Methods

### Subjects

Fourteen children with ADHD were recruited from the Children's Hospital Affiliated to Capital Institute of Pediatrics and compared with 15 TD children recruited from the local community (Table 1). Participants were group matched for age, gender, full-scale IQ, and handedness. All participants were right-handed, with an average of 6–9 years. Individuals who met the DSM-V criteria for ADHD were included in the ADHD group. IQ was evaluated using the Chinese version of the Wechsler Intelligence Scale for Children-Revised, and the IQ score of the participants was ≥70. The TD children had no history of any mental or neurological disorders. Exclusion criteria for all subjects included history of seizure or head trauma, as well as diagnosis of a neurological disorder, genetic disorder, or major medical condition. Written consent was obtained from the parents of all subjects. This study was approved by the Ethics Committee of Children's Hospital Affiliated to Capital Institute of Pediatrics.

**Table 1:** Demographic and clinical profiles for ADHD children and TD children.

**ADHD children**			**TD children**		
**ID**	**Age (years)**	**Sex**	**ID**	**Age (years)**	**Sex**
1	6	Male	1	6	Female
2	6	Male	2	6	Male
3	7	Female	3	7	Female
4	7	Female	4	7	Male
5	7	Male	5	7	Male
6	8	Female	6	7	Male
7	8	Male	7	7	Male
8	8	Male	8	8	Female
9	8	Male	9	8	Male
10	8	Male	10	8	Male
11	8	Male	11	8	Male
12	9	Male	12	9	Female
13	9	Male	13	9	Male
14	9	Female	14	9	Male
			15	9	Male
Mean	7.71			7.67	
SD	0.99			1.05	

### Experimental task

**Figure 1:**
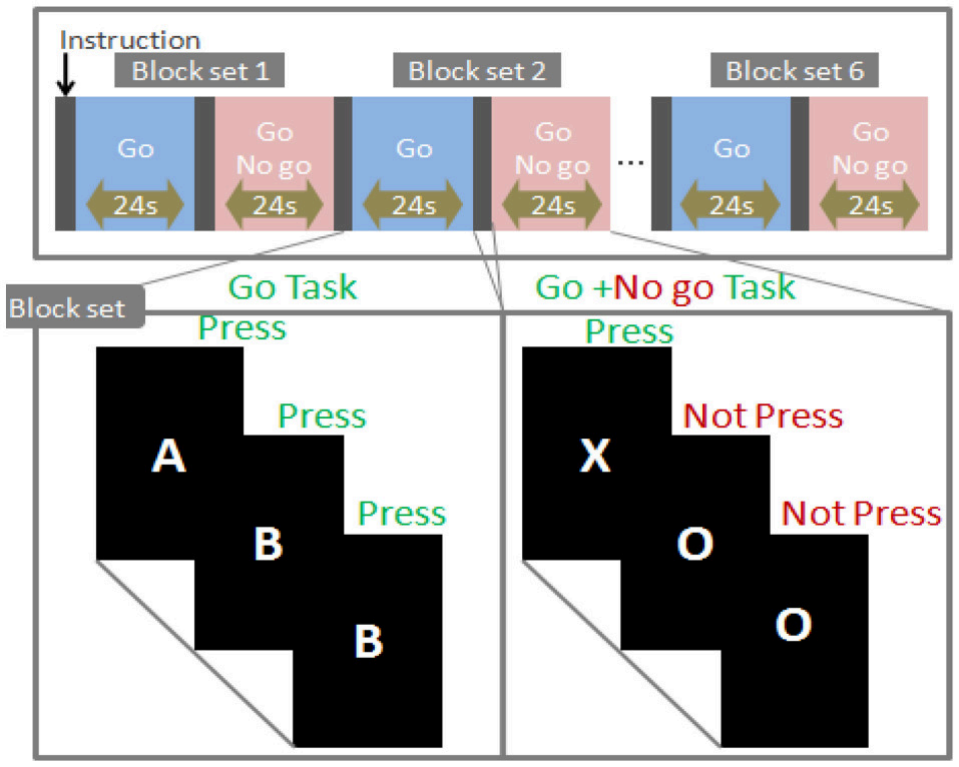
Task design.

Go/no-go task was generated by E-Prime2.0 and presented in a 17″ desktop computer screen. The distance between the subject's eyes and the screen was ~50 cm. The block-designed task consisted of six block sets (Figure 1). Each set comprised alternating go (baseline) and go/no-go (target) blocks. A 3 s instruction was presented at the beginning of each block. Each block contained 24 trials, and each trial lasted for 1 s. The entire task lasted for 5.4 min. In the go condition, subjects were presented a random sequence of two letters (“A” and “B”) and required to respond to both letters. In go/no-go blocks, participants were asked to make a response when the letter “O” was presented and inhibit their response to the letter “X.” All subjects were instructed to respond to each letter as quick as possible. The participants responded using their forefinger of the right hand. Each participant performed a practice block before any measurements to ensure that they understand the instruction. We selected a go/no-go ratio of 50% (Dillo et al., 2010; Monden et al., 2012a; Nagashima et al., 2014). The reaction time (RT) of go trials and the accuracy (ACC) for go and no-go trials were recorded.

$$\begin{array}{l} \text{ACC}=\frac{N_{r}}{N_{t}} \end{array}$$

*N*_*r*_: The number of right responses. *N*_*t*_: The total number of responses.

### fNIRS measurements

**Figure 2:**
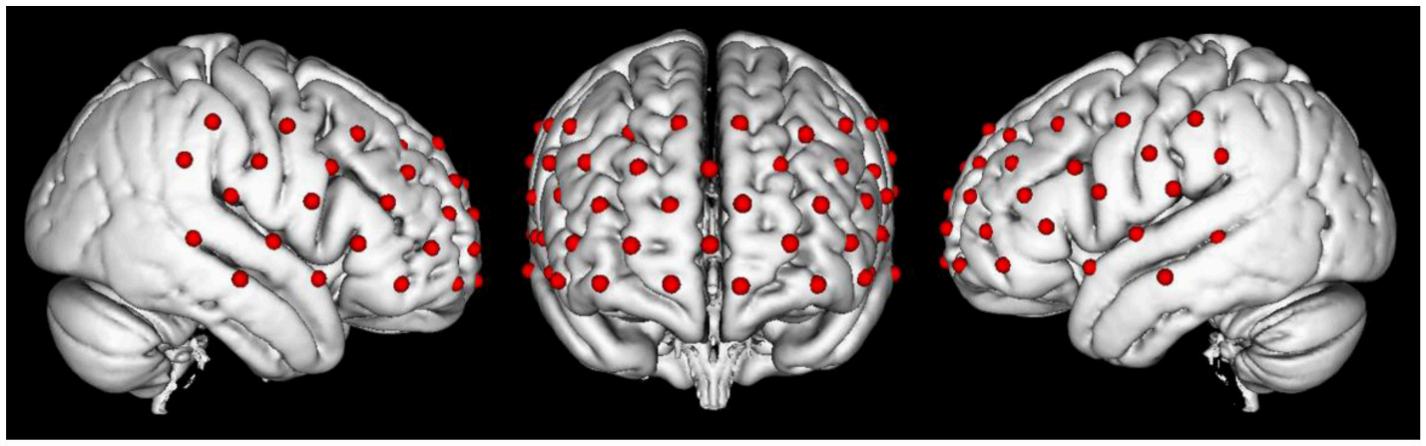
The map of fNIRS channels. Each red dot on the standard brain model represents a fNIRS channel.

Changes in the concentration of oxy-HB, deoxy-HB, and total-HB (mM.mm) was recorded in the PFC by using a continuous multichannel fNIRS instrument (ETG-4000; Hitachi Medical Corporation, Kashiwa, Japan) that worked with two different wavelengths of near-infrared light (695 and 830 nm). We utilized a probe set containing 17 sources and 16 detectors to obtain 52 fNIRS measurement channels (Figure 2). Optical data were analyzed based on the modified Beer–Lambert Law (Cope et al., 1988). The fNIRS data were measured under a sampling rate of 10 Hz. The probe-set was placed on the head with regard to the relevant standard positions of the international 10–20 system for EEG electrode placement (Klem et al., 1999; Okamoto et al., 2004). The middle inferior optode was placed on Fpz, and the inferior row of the optodes was oriented in T3 or T4 direction (Schecklmann et al., 2010).

### Analysis of fNIRS data

To analyze fNIRS data, we focused on the oxy-HB signal because of its higher sensitivity to changes in cerebral blood flow than that of deoxy-HB and total-HB (Strangman et al., 2002; Hoshi, 2003), as well as its higher signal-to-noise ratio (Strangman et al., 2002) and retest reliability (Plichta et al., 2006). The time-series data of each channel for the fNIRS data were preprocessed through filtration with a digital bandpass set between 0.01 and 0.8 Hz. A baseline correction of oxy-HB (10 s preceding the task) was carried out to compensate for drift over time. We selected relatively stable block signals without head motion and obvious noise for further analysis through visual inspection of the signals. We calculated the inter-trial mean of differences between the peak oxy-HB signals (4–24 s after go/no-go block onset) and baseline (14–24 s after go block onset) periods (Nagashima et al., 2014). To examine whether the oxy-HB change be significantly increasing in the go/no-go block relative to baseline, the average changes in oxy-HB concentration during each task minus the average changes in the baseline period before the task were determined and statistically analyzed.

### Statistical analysis

The oxy-HB signals were analyzed statistically in a channel-wise manner. First, we examined the difference between changes in oxy-HB peak and baseline for subjects with ADHD. Second, we examined the difference between changes in oxy-HB peak and baseline for the controls. Third, the difference of changes in oxy-HB peak for subjects with ADHD and control subject were calculated.

In step 1 and step 2 we examined the difference between changes in oxy-HB peak and baseline for each subject using one sample *t*-tests. To determine different brain activities between ADHD and control groups, we employed two-tailed independent sample *t*-test on the difference of changes in oxy-HB peak to identify channels involved in the go/no-go tasks.

## Results

### Behavioral performance

In the behavior data during the go/no-go task, five indices were statistically analyzed. Table 2 summarizes the average accuracy for go and no-go trials and RT for correct go trials in the go/no-go task, commission errors (response to a no-go stimulus), and omission errors (nonresponse to a Go stimulus) for controls and ADHD subjects. The results of *t*-test showed that go/no-go behavior performance was not significantly different between the control and ADHD subjects.

**Table 2:** Performance data and functional data associated with response inhibition during go/no-go task.

	**TD**		**ADHD**		***T***	***P***	
	**Mean**	***SD***	**Mean**	***SD***			
**PERFORMANCE DATA**							
RT_go trail (ms)	436.3224	52.7589	472.4019	93.0001	1.2965	0.2058	ns
ACC_go trail (%)	87.41	11.46	85.68	17.00	−0.3184	0.7528	ns
ACC_no go trail (%)	76.20	17.89	78.47	11.32	0.4045	0.6890	ns
Commission errors	17.6429	13.2119	15.3077	8.4497	−0.5422	0.5925	ns
Omission errors	9.7143	8.1564	10.3077	12.2433	0.1493	0.8825	ns
**FUNCTIONAL DATA**							
Oxy-HB CH37(mM·mm)	0.045	0.068	0.025	0.027	2.482	0.038	^*^
Oxy-HB CH48(mM·mm)	0.069	0.011	0.023	0.036	2.835	0.008	^*^

*Data are presented as mean ± SD. RT, Reaction time; TD, typical development; ns, not significant. P > 0.05*.

*, *p < 0.05*.

### fNIRS

We screened for any fNIRS channels involved in the go/no-go task for control and ADHD contrasts. We found a significant oxy-HB increase in the left CH 37(mean = 0.045, *SD* = 0.068, *p* = 0.023), 48(mean = 0.069, *SD* = 0.011, *p* = 0.002), 49 (mean = 0.051, *SD* = 0.087, *p* = 0.037) in control subjects. These channels were located in the left frontopolar cortex (FPC). But we didn't find any channels exhibited a significant oxy-HB increase in ADHD subjects.

**Figure 3:**
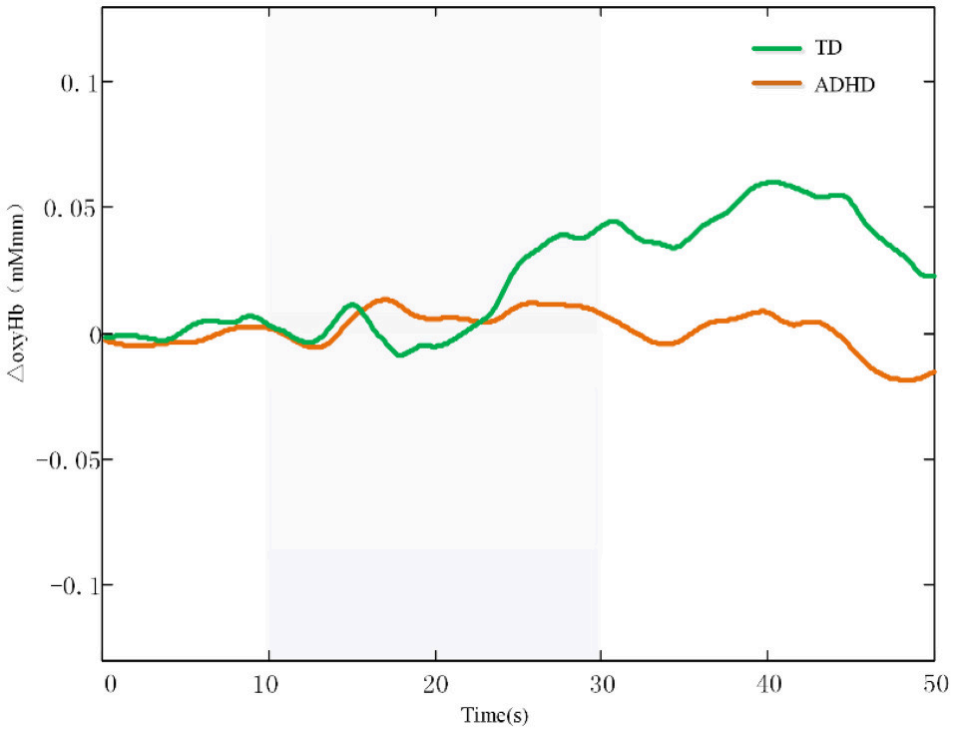
The waveforms of oxy-HB signals for CH 37. The oxy-HB signals of ADHD children is indicated in red. The oxy-HB signals of TD children is indicated in green. Oxy-HB signals is shown in units of mM·mm.

Additionally, The CH 37, CH48, and CH 49 were selected as channels of interest for investigating the difference between ADHD and TD. Comparison between oxy-HB signals of the control and ADHD subjects revealed significant activation of oxy-HB signal in the left CH 37, 48 in the control subjects (two-tailed independent sample *t*-test, Table 2). Figure 3 is the waveforms of oxy-HB signals for CH 37. These channels were located in left FPC. This finding indicates that the controls exhibited higher left FPC activation during go/no-go tasks than children with ADHD.

## Discussion

This study mainly aims to explore the feasibility of using fNIRS to differentiate children with ADHD from TD children. Left FPC activation could serve as an objective neuro-functional biomarker for fNIRS measurement. Relative to the controls, children with ADHD exhibited reduced brain activation in the left FPC during go/no-go task blocks.

### Behavioral performance for go/no-go task

The go/no-go paradigm requires response selection between executing or inhibiting a motor response as triggered by a go- or a no-go-stimulus. The task demands high-level cognitive functions of decision making, response selection, and response inhibition (Rubia et al., 2001). This cognitive function is essential in daily life, and impaired response inhibition is a potential biomarker for ADHD in children (Barkley, 1997). As such, numerous researchers investigated the disinhibitory nature of ADHD by using the go/no-go paradigm (Monden et al., 2012a; Vasic et al., 2014).

In this study, behavioral performance was not significantly different between children with ADHD and controls, similar to previously reported findings (Durston et al., 2003; Smith et al., 2006; Nagashima et al., 2014). Children with ADHD exhibit different developmental trajectories in impulse control (Barkley, 1997), and TD children show more control at the early development stage. In the present study, the participants aged 6 and 9 years. Cognitive control continues to develop over this age range (Diamond et al., 1994; Casey et al., 1997, 2001; Carver et al., 2001); thus, the divergence in developmental trajectories among groups could be the beginning in our current sample. This finding may explain the lack of differences in the overall accuracy for children who participated in the imaging study (Durston et al., 2003). However, our result is inconsistent with previous studies, in which children with ADHD manifested impaired performance compared with the controls (Monden et al., 2012a).

### fNIRS

fMRI studies on response inhibition reported frontal lobe activation (Mostofsky et al., 2003; Wager et al., 2005; Blasi et al., 2006). Therefore, in the current study, fNIRS measurements covered the PFC. We detected brain activation in the left FPC during go/no-go task blocks in TD children; moreover, fMRI studies of the Go/No-go task in TD children consistently used FPC (Casey et al., 1997; Booth et al., 2003). As such, we conclude that our current fNIRS measurements robustly extracted concurrent activation for response inhibition in the left FPC in control subjects.

Activation in the PFC was not observed during the go/no-go task period in subjects with ADHD. In addition, children with ADHD showed reduced activation in the left FPC compared with TD children. The present study further supports that children with ADHD have an inhibitory function defect. Furthermore, the left FPC function associated with the go/no-go task performance may be impaired in children with ADHD.

The left FPC dysfunction of children with ADHD in performing response inhibition tasks observed by fNIRS is consistent with other studies that employed brain imaging techniques (Smith et al., 2006; Rubia et al., 2009; Cubillo et al., 2011). In the study of Smith et al. (Smith et al., 2006), TD and ADHD children were asked to carry out go/no-go task; the fMRI results indicated that children with ADHD showed decreased activation in the left FPC during the go/no-go task. Cubillo et al. (2011) used fMRI on children with ADHD who executed response inhibition tasks (oddball task); the results showed that the level of activation of the left FPC decreased. Rubia et al. (2009) also reported the induced activation of the left FPC in ADHD children using fMRI.

FPC is the largest anterior region within the human PFC (Roca et al., 2011) and is associated with high-order cognitive functions (Badre, 2008; Vincent et al., 2008; Lee and Kim, 2014). Several researchers placed this brain region at the top of the frontal processing hierarchy (Badre and D'Esposito, 2007, 2009; Shimoda et al., 2014). Imaging studies indicated that response inhibition is highly dependent on PFC (Schmitz et al., 2006; Zang et al., 2006; Xiao et al., 2012). FPC plays a role in coordinating and integrating the dorsal lateral prefrontal cortex and ventral lateral pre-frontal cortex (Shimoda et al., 2014). It is the only PFC region that almost exclusively connected to other supramodal areas within PFC (Ramnani and Owen, 2004; Burgess et al., 2007). Furthermore, the FPC area can control sustained attention (Sturm and Willmes, 2001; Derosiere et al., 2014). Researchers assumed that reduced FPC activation during intact inhibitory performance may be related to comeasured processes of selective attention and decision making (Rubia et al., 2003; Smith et al., 2006; Monden et al., 2012b). Furthermore, several investigators believed that a high go/no-go ratio may lead to activation during no-go blocks and is associated with selective attention rather than response inhibition (Tamm et al., 2004; Dillo et al., 2010; Monden et al., 2012b). By contrast, a go/no-go ratio of 50% was selected because it is commonly used in neuroimaging studies (Tamm et al., 2004; Dillo et al., 2010; Monden et al., 2012b).

fNIRS study also added further evidence regarding the involvement of the left PFC during go/no-go tasks. In the study of Fangyue, children with ADHD showed weaker activation and impaired cognitive function in the left PFC than TD children (Fang et al., 2010). Moreover, recent fNIRS study reported reduced prefrontal activation in children with ADHD compared with normal controls during a go/no-go condition (albeit no laterality was reported; Inoue et al., 2012). In addition, several fNIRS studies observed that ADHD children showed reduced activation during go/no-go task in the right 56 middle frontal cortex (MFC)/inferior frontal cortex (IFC) region (Monden et al., 2012a). Hence, differences between studies in go/no-go task designs and contrast conditions may explain differences in laterality or precise localization (Rubia et al., 2001). These data illustrate that fNIRS technique can be used to investigate cerebral hemodynamic in ADHD during response inhibition tasks.

## Limitations

This study has several limitations, which include a small sample size and fNIRS measurement. The sample size in the present study is rather small, thereby limiting our ability to detect subtle differences among groups. Therefore, future studies must have large sample size to confirm our conclusions. Given that the fNIRS system could cover the PFC only, we did not examine any other cortical areas, except PFC. Moreover, fNIRS cannot detect the activities of deep sub-cortical structures where near-infrared light cannot reach. Hence, a wider range of cortex should be included in further study. Furthermore, this technique must be combined with other imaging methods to investigate relationships between PFC activity and stimulus responses.

## Conclusion

In this study, we monitored prefrontal cortex activation through fNIRS of children with ADHD and TD children who performed a go/no-go task (response inhibition task). We obtained the following findings: First, activation foci (left FPC) were activated in TD children who performed a go/no-go task only. Second, relative to control subjects, children with ADHD exhibited reduced brain activation in the left FPC during go/no-go task blocks. Hence, left PFC activation could be an objective neuro-functional biomarker to distinguish children with ADHD and TD children. fNIRS-based examination on ADHD assisted diagnosis is applicable to children at elementary school ages, including those as young as 6 years old. Therefore, fNIRS-based examination is a promising clinical tool for early diagnosis of patients with ADHD.

## Ethics statement

This study was carried out in accordance with the recommendations of the Ethics Committee of Capital Institute of Pediatrics with written informed consent from all subjects. All subjects gave written informed consent in accordance with the Declaration of Helsinki. The protocol was approved by the Ethics Committee of Capital Institute of Pediatrics.

## Author contributions

SM: Experimental design, data collection, paper writing. JH and YG: Test task writing, data processing. XW, WS, DL and ZL: Data collection. JY and XL: Experimental design, Project implementation management.

### Conflict of interest statement

The authors declare that the research was conducted in the absence of any commercial or financial relationships that could be construed as a potential conflict of interest.
